# Supplementary material for: Information-to-work conversion by Maxwell’s demon in a superconducting circuit quantum electrodynamical system
Source: Nat Commun. 2018 Mar 29;9:1291. doi: 10.1038/s41467-018-03686-y (PMC5876355; doi:10.1038/s41467-018-03686-y)
Supplement: Supplementary file 1 — Supplementary Information(PDF 853 kb) [file 41467_2018_3686_MOESM1_ESM.pdf]

Supplementary Information for "Information-to-work conversion by Maxwell's demon in a superconducting circuit quantum electrodynamical system"

Y. Masuyama *et al.*

Supplementary Note 1. GENERALIZED INTEGRAL FLUCTUATION THEOREM

We derive the generalized integral fluctuation theorem under feedback control [1] in the context of our experimental protocol. Figure 1 illustrates an example of a feedback process for a qubit. We perform a quantum-non-demolition (QND) projective measurement on the initial canonical state of the qubit

$$\hat{\rho}_{\rm ini} = \sum_{x={\rm g,e}} p_{\rm can}(x)|x\rangle\langle x|, \tag{1}$$

and obtain the state $\hat{\rho}_x = |x\rangle\langle x|$ characterized by the measurement outcome x with probability $p_{\rm can}(x)$ [Fig. 1(i)]. Here $|{\rm g}\rangle$ and $|{\rm e}\rangle$ are the qubit energy eigenstates. Next, we perform a measurement (outcome k) for the feedback control [Fig. 1(ii)]. Due to the backaction of this measurement, the post-measurement state becomes

$$\hat{\rho}_k = \frac{\hat{M}_k \hat{\rho}_x \hat{M}_k^\dagger}{p(k)}, \tag{2}$$

where $\hat{M}_k$ and $p(k)$ denote the Kraus operator describing the measurement process and the probability for the outcome k, respectively. By the subsequent projective measurement (outcome y) [Fig. 1(iii)], one obtains the stochastic QC-mutual information

$$I_{\rm QC}(x,k,y) = \ln p(y|k) - \ln p_{\rm can}(x), \tag{3}$$

where $p(y|k)$ is the probability of the outcome y conditioned on the outcome k. We perform a feedback operation $\hat{U}_k$, which depends on the measurement outcome k for the feedback control, on the state $\hat{\rho}_k(y) = |y\rangle\langle y|$ [Fig. 1(iv)]. Subsequently, we perform a projective measurement (outcome z) and obtain the final state $\hat{\rho}_k(z) = |z\rangle\langle z|$ [Fig. 1(v)]. The probability distribution of the forward process is

$$p(x,k,y,z) = p_{\rm can}(x)\, p(k,y\,|\,x)\, p(z\,|\,k,y), \tag{4}$$

where $p(k,y\,|\,x) = |\langle y|\hat{M}_k|x\rangle|^2$ and $p(z\,|\,k,y) = |\langle z|\hat{U}_k|y\rangle|^2$.

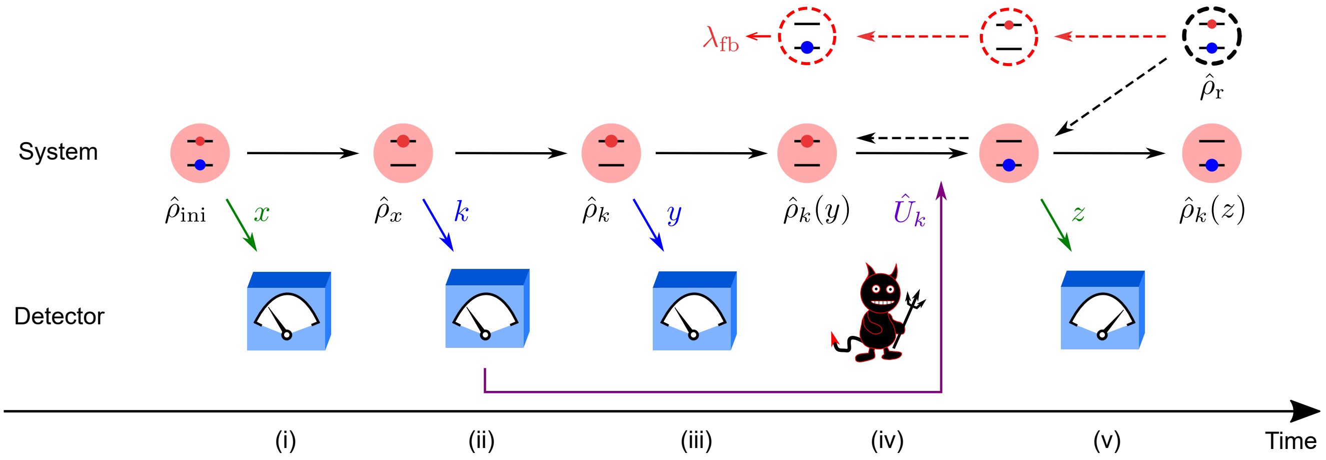

Supplementary Figure 1. Schematic illustration of a feedback process of a qubit. (i) Projective measurement of the initial state $\hat{\rho}_{\rm ini}$. Here, for simplicity, we illustrate the case where the initial state x of the qubit is found to be in the excited state. (ii) Measurement for the feedback control. The measurement outcome k is recorded in the detector, and a feedback operation [i.e., (iv)] is later performed based on the outcome. (iii) Projective measurement of the state $\hat{\rho}_k$ right after the measurement for the feedback control. When the measurement in (ii) is a quantum non-demolition projective measurement on the qubit eigenbasis, the post-measurement state y should be the excited state, and therefore the probability of being found in the ground state (dashed circle) vanishes. (iv) Feedback operation based on the outcome k. As k indicates that the state is in the excited state, a π-pulse is applied to flip the qubit. (v) Measurement of the final state. The final state z is found to be in the ground state. In the time-reversed process from the reference state $\hat{\rho}_{\rm r}$ in the canonical distribution, the system may evolve into the ground state as indicated by the red dashed arrow. This event has no counterpart in the forward process, and therefore contributes to the degree of absolute irreversibility denoted by $\lambda_{\rm fb}$ which is calculated as the total probability of such transitions.

Next we introduce the time-reversed process to derive the fluctuation theorem for the entropy production $\sigma = -\beta(W + \Delta F)$, where W denotes the extracted work and ΔF is the free-energy difference. In the present situation, since there is no difference between the initial and final Hamiltonians, ΔF vanishes. The initial state of the time-reversed process, which is called the reference state $\hat{\rho}_{\mathrm{r}}$, is chosen to be the same canonical distribution as the initial state

$$\hat{\rho}_{\mathrm{r}} = \sum_{z=\mathrm{g,e}} p_{\mathrm{can}}(z)|z\rangle\langle z|. \tag{5}$$

We define the probability distribution under the time-reversed feedback operation as $\tilde{p}(k, y, z) = \tilde{p}(y\,|\,k, z)\, p(k)\, p_{\mathrm{can}}(z)$, where we define the time-reversed transition probability as $\tilde{p}(y\,|\,k, z) := |\langle y|\hat{U}_k^{\dagger}|z\rangle|^2 = p(z\,|\,k, y)$. The fluctuation theorem can be derived by forming the ratio of the time-reversed probability $\tilde{p}(k, y, z)$ to the forward probability $p(k, y, z) := \sum_x p(x, k, y, z)$. To do so, we should separate the set $Y = \{y\,|\,p(y|k) \neq 0\}$ to guarantee that the denominator of the ratio does not vanish. Then, the fluctuation theorem is derived as follows:

$$\begin{aligned}
1 &= \sum_{k,y\in Y,z} \tilde{p}(k, y, z) + \sum_{k,y\notin Y,z} \tilde{p}(k, y, z) \\
&= \sum_{x,k,y\in Y,z} p(x, k, y, z)\, \frac{\tilde{p}(k, y, z)}{p(k, y, z)} + \sum_{k,y\notin Y,z} \tilde{p}(k, y, z) \\
&= \sum_{x,k,y\in Y,z} p(x, k, y, z)\, \mathrm{e}^{\beta W(x,z) - I_{\mathrm{QC}}(x,k,y)} + \sum_{k,y\notin Y,z} \tilde{p}(k, y, z) \\
&= \langle \mathrm{e}^{\beta W(x,z) - I_{\mathrm{QC}}(x,k,y)} \rangle + \sum_{k,y\notin Y,z} \tilde{p}(k, y, z),
\end{aligned} \tag{6}$$

where we use Eq.(3) and $-\beta W(x, z) = \sigma(x, z) = \ln[p_{\mathrm{can}}(x)/p_{\mathrm{can}}(z)]$ to obtain the third line. For the events with $y \notin Y$, the entropy production formally diverges due to the detailed fluctuation theorem [2, 3] as $\ln(0/p) = -\infty$. These events with divergent entropy production (red dotted line in Fig. 1) are called absolutely irreversible events [1]. To circumvent the problem of divergence, one should subtract the total probability of the absolutely irreversible events defined by

$$\lambda_{\mathrm{fb}} := \sum_{k,y\notin Y,z} \tilde{p}(k, y, z). \tag{7}$$

As a result, we obtain the generalized integral fluctuation theorem under feedback control in the presence of absolute irreversibility [1]:

$$\langle \mathrm{e}^{\beta W - I_{\mathrm{QC}}} \rangle = 1 - \lambda_{\mathrm{fb}}. \tag{8}$$

By applying Jensen's inequality, we obtain

$$\beta\langle W\rangle \leq \langle I_{\mathrm{QC}}\rangle + \ln(1 - \lambda_{\mathrm{fb}}). \tag{9}$$

The inequality (9) can be regarded as the generalized second law of thermodynamics that incorporates the effects of feedback control.

If the readout k for the feedback control is not a projective readout, λ_{fb} vanishes and Eq.(8) reduces to $\langle \mathrm{e}^{\beta W - I_{\mathrm{QC}}} \rangle = 1$ as demonstrated in Fig. 3(b). On the other hand, I_{QC} reduces to the stochastic Shannon entropy I_{Sh} when the measurement is a projective measurement as in the experiment shown in Fig. 2. In this case, the left-hand side of the generalized integral fluctuation theorem can be calculated from the experimentally accessible values as

$$\begin{aligned}
\langle \mathrm{e}^{\beta W - I_{\mathrm{Sh}}} \rangle_{\mathrm{PM}} &= \sum_{x,z} p(x, z)\, \mathrm{e}^{\beta W(x,z) - I_{\mathrm{Sh}}(x)} \\
&= p(x = \mathrm{g})\, p(z = \mathrm{e}\,|\,x = \mathrm{g})\, \mathrm{e}^{-\beta\hbar\omega_{\mathrm{q}} - I_{\mathrm{Sh}}(x=\mathrm{g})} + p(x = \mathrm{e})\, p(z = \mathrm{g}\,|\,x = \mathrm{e})\, \mathrm{e}^{\beta\hbar\omega_{\mathrm{q}} - I_{\mathrm{Sh}}(x=\mathrm{e})} \\
&\quad + p(x = \mathrm{e})\, p(z = \mathrm{e}\,|\,x = \mathrm{e})\, \mathrm{e}^{-I_{\mathrm{Sh}}(x=\mathrm{e})} + p(x = \mathrm{g})\, p(z = \mathrm{g}\,|\,x = \mathrm{g})\, \mathrm{e}^{-I_{\mathrm{Sh}}(x=\mathrm{g})},
\end{aligned} \tag{10}$$

where $p(x)$ $(x = \mathrm{g, e})$ is the probability of observing the state x in the first measurement of the two-point measurement protocol, the inverse temperature $\beta = (\hbar\omega_{\mathrm{q}})^{-1} \ln\left[p(x = \mathrm{g})/p(x = \mathrm{e})\right]$, and $p(z\,|\,x)$ is the conditional probability of observing the state z $(z = \mathrm{g, e})$ in the second measurement after observing the state x in the first one. Moreover, $I_{\mathrm{Sh}}(x) = -\ln p(x)$ denotes the stochastic Shannon information obtained when the state x is observed. The notation $\langle\cdot\rangle_{\mathrm{PM}}$ indicates a statistical average obtained in a protocol with a projective measurement. In this case, the maximum value of the extracted work is determined by $\beta\langle W\rangle_{\mathrm{PM}} \leq \langle I_{\mathrm{Sh}}\rangle_{\mathrm{PM}} + \ln(1 - \lambda_{\mathrm{fb}})$. When the equality is achieved, the maximum feedback efficiency with this feedback protocol is also achieved [see the inset in Fig. 3(c)].

Supplementary Note 2. EXPERIMENTAL DETAILS

Cryogenic environment

The experiment was conducted in a cryogen-free dilution refrigerator with the base temperature of about 10 mK. The cavity enclosing the supeconducting qubit is placed inside a magnetic shield. A flux-driven Josephson parametric amplifier (JPA) [4] is placed in a separated magnetic shield and is biased with a small solenoid at a static magnetic field as well as pumped at twice of the resonance frequency. The probe pulse for the readout is introduced to the cavity through a series of attenuators, and the reflected signal is amplified by the JPA operated in the degenerate mode and by the following amplifiers at 4-K and 300-K stages (Fig. 2).

Sample

The circuit quantum-electrodynamical system is constructed with a transmon-type superconducting qubit [5] in a cavity.

The transmon qubit is fabricated on a sapphire substrate. The size of the two aluminum pads of the transmon is $250 \times 500\ \mu\mathrm{m}^2$ each, and the area of the Josephson junction is $150 \times 250\ \mathrm{nm}^2$. The qubit has the bare resonance frequency $\omega_{\mathrm{q}}/2\pi = 6.6296$ GHz and the anharmonicity of -345 MHz. The latter is defined as the difference between the excitation frequencies from the ground to the first excited states and from the first to the second excited states. From independent time-domain experiments, we obtained the energy relaxation time $T_1 = 24\ \mu$s, and the phase relaxation time $T_2^* = 16\ \mu$s.

The aluminum-made rectangular cavity has the fundamental mode TE_{101} with the bare resonance frequency of $\omega_{\mathrm{cav}}/2\pi = 10.6180$ GHz, largely detuned from the qubit. It has a single SMA-connector port, and the relaxation time $1/\kappa = 0.076\ \mu$s is determined by the sum κ of the external and internal loss rates, $\kappa_{\mathrm{ex}}/2\pi = 1.47$ MHz and $\kappa_{\mathrm{in}}/2\pi = 0.63$ MHz, with the qubit mounted inside.

The qubit is mounted at the center of the cavity. The coupling strength between the qubit and the cavity mode is estimated to be $g/2\pi = 0.14$ GHz from the measurement of the dispersive shift $\chi/2\pi = (g^2/\Delta)/2\pi = -4.6$ MHz of the cavity mode, where $\Delta = \omega_{\mathrm{cav}} - \omega_{\mathrm{q}}$ is the detuning between the qubit and the cavity. Due to the interaction, the cavity

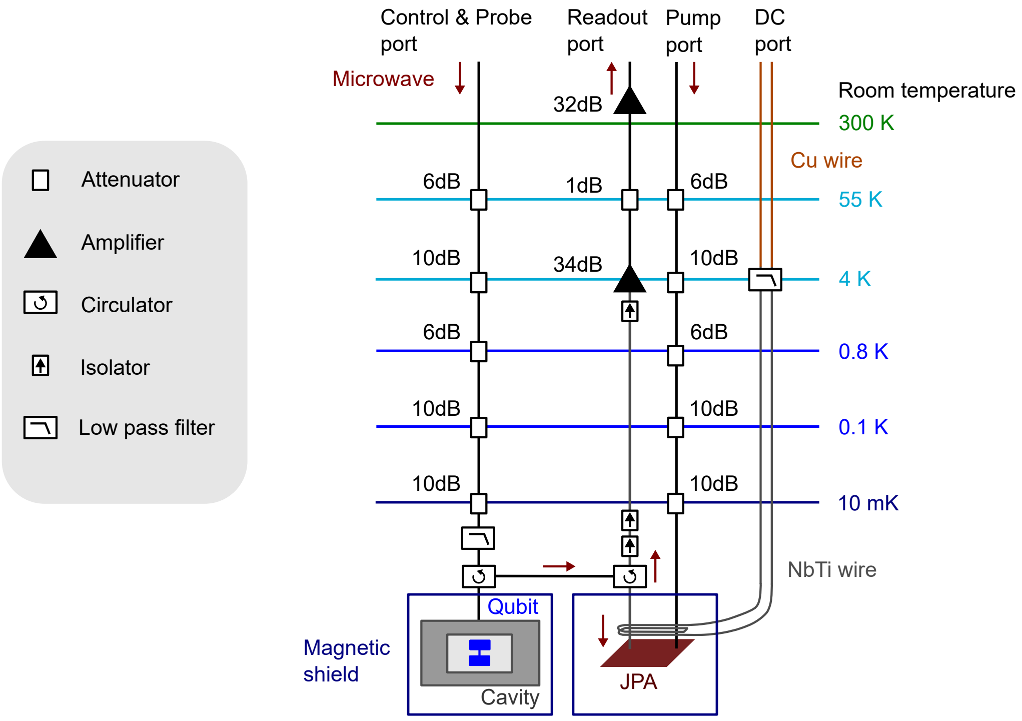

Supplementary Figure 2. Schematic of the wiring in the dilution refrigerator.

frequency is shifted to $(\omega_{\rm cav} + g^2/\Delta)/2\pi = 10.6226$ GHz, and the qubit frequency is shifted to $(\omega_{\rm q} - g^2/\Delta)/2\pi = 6.6342$ GHz.

Quantum non-demolition projective readout of the qubit

In the dispersive regime where the detuning Δ is much larger than the qubit-cavity coupling strength g, the Jaynes-Cummings Hamiltonian of the coupled system can be approximated as [6]

$$\begin{aligned} H_{\rm JC} &= \hbar\omega_{\rm cav}\left(\hat{a}^\dagger\hat{a} + \frac{1}{2}\right) + \frac{\hbar\omega_{\rm q}}{2}\hat{\sigma}_z + \hbar g\left(\hat{a}^\dagger\hat{\sigma}_- + \hat{a}\hat{\sigma}_+\right) \\ &\approx \hbar\omega_{\rm cav}\left(\hat{a}^\dagger\hat{a} + \frac{1}{2}\right) + \frac{\hbar}{2}\left(\omega_{\rm q} - \frac{g^2}{\Delta}\right)\hat{\sigma}_z - \hbar\frac{g^2}{\Delta}\hat{\sigma}_z\hat{a}^\dagger\hat{a}, \end{aligned} \tag{11}$$

where $\hat{a}$ is the annihilation operator of the cavity mode, $\hat{\sigma}_z$ is the Pauli operator of the qubit, and $\hat{\sigma}_+$ and $\hat{\sigma}_-$ are the qubit raising and lowering operators, respectively. It indicates that the cavity resonance frequency depends on the states of the qubit, giving rise to the so-called dispersive shift. Thus, the states of the qubit can be projected onto the energy eignenstates by the measurement of a phase shift of a resonant microwave pulse reflected by the cavity [6].

As the interaction term, i.e., the last term in Eq.(11), commutes with the qubit Hamiltonian $\propto \hat{\sigma}_z$, the dispersive readout has a quantum non-demolition nature, which is crucial in the present work.

Experimental setup for the qubit readout

We generate the microwave probe pulses for qubit readout by using single-side-band modulation of the continuous carrier microwaves with a 50-MHz intermediate-frequency (IF) signal from a digital-to-analog converter (DAC; UCSB GHzDAC, 1 GSa/s, 12-bit resolution). The temporal shape of the pulse, i.e., the amplitude and the phase, is defined by multiplying the waveform from the DAC and the carrier microwaves at an IQ mixer. We adjust the the microwave carrier frequency of the readout pulses at 10.6219 GHz to obtain the largest difference in the complex amplitude of the electric field between the reflection signals corresponding to the ground state and the excited state of the qubit.

The readout signal, reflected by the cavity and amplified in the chain of the amplifiers in Fig. 2, passes through a frequency-tunable resonator-type bandpass filter (bandwidth 50 MHz) and is down-converted to the intermediate frequency of 50 MHz with an IQ mixer and a local oscillator (Fig. 4). One of the IF ports of the IQ mixer is connected to an analog-to-digital converter (ADC; Acqiris AP240, 1 GSa/s, 8-bit resolution) for signal acquisitions in the readouts x, y and z (not shown in the figure), and the other is connected to the feedback system described below.

The signal acquired in the ADC is digitally processed to discriminate the ground and the excited states. The separation of the signals for the 500-ns-wide projective readout pulse is almost 100% (Fig. 3). Moreover, we checked the quantum non-demolition property of the measurement by two subsequent measurements of the qubit. In the test with two 1-μs-wide readout pulses, 99.6% of the ground state observed in the first measurement remained in the ground state at the second measurement, and 96.6% of the excited state stayed in the same state. The amount of the reductions can be attributed to the energy relaxation of the qubit during the interval (about 0.3 μs) between the

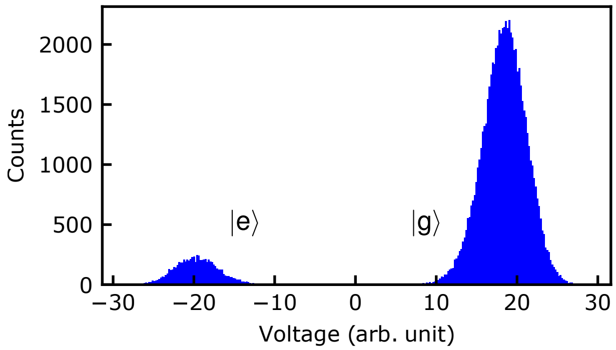

Supplementary Figure 3. An example of the histogram of the readout outcomes.

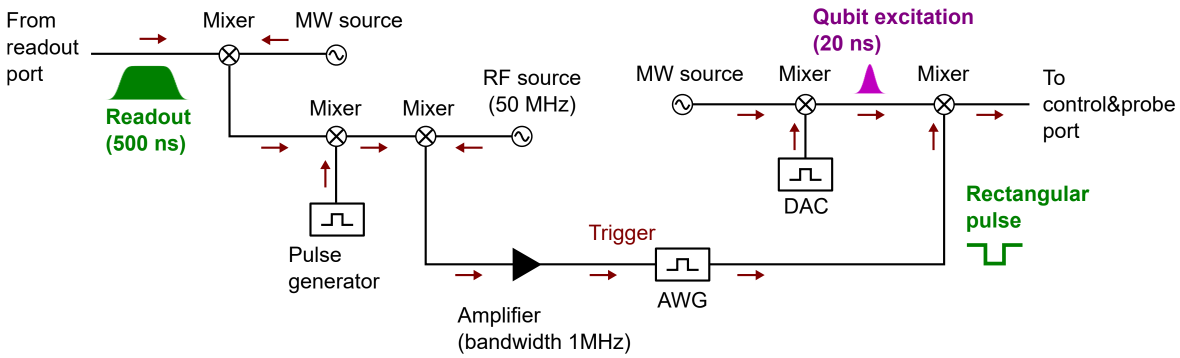

Supplementary Figure 4. Experimental setup for the feedback control.

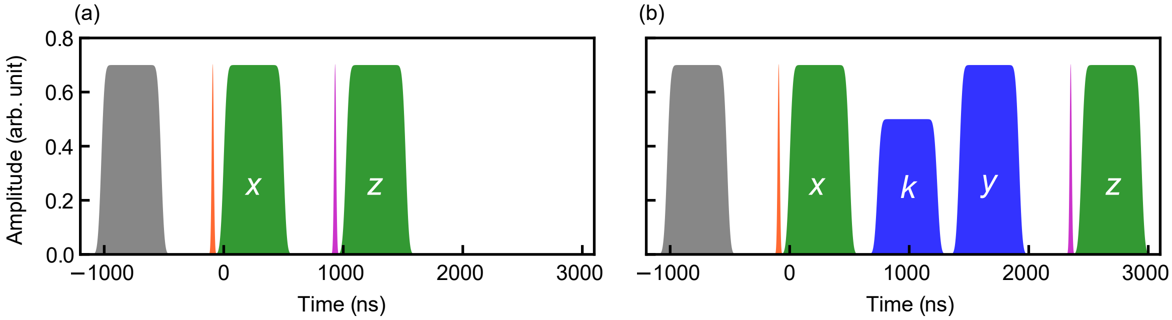

Supplementary Figure 5. Details of the pulse sequences. The origin of the time is set at the beginning of the two-point measurement protocol. (a) Pulse sequence used in the experiment in Fig. 2. (b) Pulse sequence used in the experiment of Fig. 3. The color codes and labels (x, y, z, k) are: readout pulse for initialization (gray), qubit excitation pulse (orange), projective readout pulses for the two-point measurement protocol (x, z), π-pulse for feedback control (magenda), and variable-amplitude readout pulse (k) and subsequent projective readout pulse (y) (blue).

pulses. This gives an upper limit of the error in the evaluation of the temperatures in Fig. 2b of the main text. The amount of the error is estimated to be at most a few percent and smaller than the size of the data points.

Experimental setup for the feedback control

Figure 4 illustrates the feedback system used in the experiment. A π-pulse is applied to the qubit only if the readout for the feedback control finds the qubit in the excited state.

As shown in Fig. 4, the down-converted readout signal at the IF frequency is first chopped at a mixer with a rectangular pulse from a pulse generator (Stanford Research Systems DG535) to select the time window used in the following analog processing, and is further down-converted to zero frequency. The obtained pulse signal is amplified with an amplifier (NF Corporation N5307, gain $\times 20$, 1-MHz filter bandwidth), and is input to the trigger port of an arbitrary waveform generator (AWG; Tektronix AWG430). The threshold of the trigger is adjusted such that the AWG outputs a negative pulse on top of the positive offset voltage only for the input signal corresponding to the qubit ground state. The triggered (untriggered) events are recorded as the outcome $k = \mathrm{g}$ $(k = \mathrm{e})$.

The π-pulse for the feedback control is generated by single-sideband modulation of the continuous microwaves at the qubit drive frequency with a DAC. Finally, the voltage pulse from the AWG suppresses the output of the π-pulse when it is triggered. The total delay in the feedback control is about 200 ns measured from the end of the readout pulse.

Details of the pulse sequences

Figure 5 illustrates the detailed timings of the pulse sequences. The qubit readout pulses are 500-ns wide with Gaussian-shaped rise and fall edges of 52-ns wide. The inverse temperature of the qubit, β, is determined by the first readout in the two-point measurement protocol by assuming the Boltzmann distribution. In the absence of the initialization readout and the excitation pulse, the effective temperature of the qubit equilibrated with the cavity field is found to be about 0.16 K, which is significantly higher than the refrigerator temperature presumably because of the residual noise introduced through the microwave cables.

The qubit control pulse has a Gaussian shape with the width of 20 ns and is applied after the preceding readout pulse with a waiting time longer than the cavity decay time to avoid photons in the cavity injected by the readout pulse causing a Stark shift of the qubit resonant frequency.

Error probability in feedback control

Here, we consider the error probability in the feedback control. Whether or not to inject the π-pulse as the feedback control is determined by the variable-strength readout (outcome k) in Fig. 3, while the state of the qubit after the readout is immediately confirmed by the strong measurement (outcome y). Events in which k and y are not the same are counted as errors of the feedback control. Let $n(y=\mathrm{g},\,k=\mathrm{e})$ [$n(y=\mathrm{e},\,k=\mathrm{g})$] denote the number of events, where the π-pulse is (not) injected erroneously when the qubit state observed as y is the ground state (the first excited state). Let n_{all} denote the total number of the repeated postselected sequences; then the probabilities for these errors can be written as [see Fig. 6(a)]

$$\epsilon(y=\mathrm{g},\,k=\mathrm{e}) := \frac{n(y=\mathrm{g},\,k=\mathrm{e})}{n_{\mathrm{all}}}, \tag{12}$$

$$\epsilon(y=\mathrm{e},\,k=\mathrm{g}) := \frac{n(y=\mathrm{e},\,k=\mathrm{g})}{n_{\mathrm{all}}}. \tag{13}$$

The error probability of the feedback control in Fig. 3 is defined as

$$\epsilon_{\mathrm{fb}} := \epsilon(y=\mathrm{g},\,k=\mathrm{e}) + \epsilon(y=\mathrm{e},\,k=\mathrm{g}). \tag{14}$$

The feedback operations with these errors are modeled as

$$\hat{I}' := \sqrt{1-\epsilon(y=\mathrm{g},\,k=\mathrm{e})}\,\hat{I} + \sqrt{\epsilon(y=\mathrm{g},\,k=\mathrm{e})}\,\hat{\sigma}_x, \tag{15}$$

$$\hat{U}'_{\pi} := \sqrt{\epsilon(y=\mathrm{e},\,k=\mathrm{g})}\,\hat{I} + \sqrt{1-\epsilon(y=\mathrm{e},\,k=\mathrm{g})}\,\hat{\sigma}_x. \tag{16}$$

The probability $1-\lambda_{\mathrm{fb}}$ that the time-reversed events have their counterparts in the forward process under the feedback control is evaluated from Eq.(7) and the first line of Eq.(6) as

$$\begin{aligned} 1-\lambda_{\mathrm{fb}} &= \mathrm{Tr}[p(k=\mathrm{g})\,\langle \mathrm{g}|\hat{I}'^{\dagger}\hat{\rho}_{\mathrm{r}}\hat{I}'|\mathrm{g}\rangle] + \mathrm{Tr}[p(k=\mathrm{e})\,\langle \mathrm{e}|\hat{U}'^{\dagger}\hat{\rho}_{\mathrm{r}}\hat{U}'|\mathrm{e}\rangle] \\ &= p(k=\mathrm{g})\,[p_{\mathrm{can}}(\mathrm{g})\,\{1-\epsilon(y=\mathrm{g},\,k=\mathrm{e})\} + p_{\mathrm{can}}(\mathrm{e})\,\epsilon(y=\mathrm{g},\,k=\mathrm{e})] \\ &\quad + p(k=\mathrm{e})\,[p_{\mathrm{can}}(\mathrm{e})\,\epsilon(y=\mathrm{e},\,k=\mathrm{g}) + p_{\mathrm{can}}(\mathrm{g})\,\{1-\epsilon(y=\mathrm{e},\,k=\mathrm{g})\}], \end{aligned} \tag{17}$$

where $p(k=\mathrm{g})$ and $p(k=\mathrm{e})$ are the probabilities of observing the measurement outcomes $k=\mathrm{g}$ and $k=\mathrm{e}$ in the forward process, respectively. The initial state of the time-reversed process is the canonical state $\hat{\rho}_{\mathrm{r}} = p_{\mathrm{can}}(\mathrm{g})|\mathrm{g}\rangle\langle\mathrm{g}| + p_{\mathrm{can}}(\mathrm{e})|\mathrm{e}\rangle\langle\mathrm{e}| = \frac{1}{Z}\left(|\mathrm{g}\rangle\langle\mathrm{g}| + \mathrm{e}^{-\beta\hbar\omega_{\mathrm{q}}}|\mathrm{e}\rangle\langle\mathrm{e}|\right)$, where Z is the partition function.

To characterize the origins of the error more precisely, we define the conditional error probabilities of the feedback control as

$$\epsilon(k=\mathrm{e}\,|\,y=\mathrm{g}) := \frac{\epsilon(y=\mathrm{g},\,k=\mathrm{e})}{p(y=\mathrm{g})}, \tag{18}$$

$$\epsilon(k=\mathrm{g}\,|\,y=\mathrm{e}) := \frac{\epsilon(y=\mathrm{e},\,k=\mathrm{g})}{p(y=\mathrm{e})}. \tag{19}$$

Note that the probabilities are conditioned on outcome y. As shown in Fig. 6(b), these errors are slightly asymmetric. There are two reasons for the asymmetry. One is the relaxation of the qubit occurring between the two readouts for k and y, which is dominant for the strong measurement. The other is the small offset in the threshold voltage for discriminating the readout signal, which was slightly biased in favour of signalling the excited state. In the case of the weak readout pulse amplitude, the error probability $\epsilon(k=\mathrm{e}\,|\,y=\mathrm{g})$ is larger than $\epsilon(k=\mathrm{g}\,|\,y=\mathrm{e})$ due to the offset.

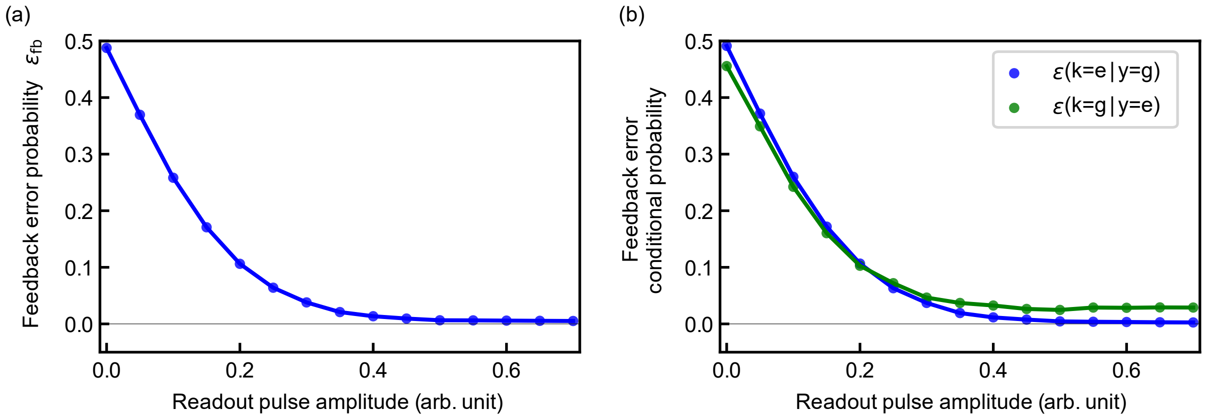

Supplementary Figure 6. Feedback error probability vs. readout pulse amplitude. The effective temperature of the initial state of the qubit in this measurement and the experiment of Fig. 3 is prepared at 0.14 K (the occupation probability of the excited state is 0.097). (a) Feedback error probability $\epsilon_{\rm fb}$. (b) Conditional error probabilities $\epsilon(k = {\rm e} \,|\, y = {\rm g})$ (blue) and $\epsilon(k = {\rm g} \,|\, y = {\rm e})$ (green).

Quantum trajectory method

The quantum trajectory method (Monte Carlo wave function method) is one of the methods to calculate the relaxation of a small quantum system coupled to a heat bath [7, 8]. We calculate the temporal evolution of the quantum system using a state vector (wave function) and a non-Hermitian Hamiltonian. It is equivalent to the calculation based on the quantum master equation [9], but has an advantage that it enables explicit calculation of each trajectory of the system evolution.

The state vector after an infinitesimal time δt is calculated from the state vector $|\Psi(t)\rangle$ of the qubit at time t by using the non-Hermitian Hamiltonian

$$\hat{H} = \hat{H}_{\rm s} - \frac{i\hbar}{2} \sum_{k=0,1} \hat{L}_k^\dagger \hat{L}_k, \tag{20}$$

where $\hat{H}_{\rm s}$ is the system Hamiltonian and $\hat{L}_k$ is the Lindblad operator. Here we ignore the effect of dephasing and only consider the relaxation which is relevant to the protocols in the present work. Thus, we use $\hat{L}_0 = \sqrt{\Gamma_\downarrow}\hat{\sigma}^-$, and $\hat{L}_1 = \sqrt{\Gamma_\uparrow}\hat{\sigma}^+$, where $\Gamma_\uparrow$ and $\Gamma_\downarrow$ are the excitation and relaxation rates of the qubit, respectively, and $\hat{\sigma}^+ = (\hat{\sigma}^-)^\dagger$ is the raising operator of the qubit. The state vector at time $t + \delta t$ is obtained as

$$\begin{aligned} |\Psi'(t+\delta t)\rangle &= \exp(-i\hat{H}\delta t/\hbar)|\Psi(t)\rangle \\ &\approx \left(1 - \frac{i\hat{H}\delta t}{\hbar}\right)|\Psi(t)\rangle. \end{aligned} \tag{21}$$

Since the above state vector is obtained by applying the non-Hermitian operator, it is not normalized. Up to the leading order in δt, the norm of the state vector is given as $\langle\Psi'(t+\delta t)|\Psi'(t+\delta t)\rangle \approx 1 - \delta p$, where we define

$$\delta p = \delta t \frac{i}{\hbar} \langle\Psi(t)| \left(\hat{H} - \hat{H}^\dagger\right) |\Psi(t)\rangle = \sum_{k=0,1} \delta p_k \tag{22}$$

$$\delta p_k = \delta t \langle\Psi(t)|\hat{L}_k^\dagger \hat{L}_k|\Psi(t)\rangle \geq 0. \tag{23}$$

Here, $\delta p = \delta p_0$ when the qubit is in the excited state and $\delta p = \delta p_1$ when it is in the ground state. Then, to decide whether a quantum leap occurs or not, a random number ϵ uniformly distributed from 0 to 1 is selected at each time step and compared with δp.

If $\delta p < \epsilon$, the state evolves to

$$|\Psi(t+\delta t)\rangle = \frac{|\Psi'(t+\delta t)\rangle}{\sqrt{1-\delta p}}. \tag{24}$$

On the other hand, if $\delta p > \epsilon$, the state jumps to a new state vector. The probability of choosing an individual state vector $\hat{L}_k|\Psi(t)\rangle$ is $\delta p_k/\delta p$. After the normalization, the new state vector is written as

$$|\Psi(t+\delta t)\rangle = \frac{\hat{L}_k|\Psi(t)\rangle}{\sqrt{\langle\Psi(t)|\hat{L}_k^\dagger \hat{L}_k|\Psi(t)\rangle}} = \frac{\hat{L}_k|\Psi(t)\rangle}{\sqrt{\delta p_k/\delta t}}. \quad (25)$$

We apply the above method in the numerical calculations of the qubit state evolutions and those of the measurement outcomes under the experimental protocols of Fig. 5. The outcome of each readout, g or e, is evaluated from the sign of the time-averaged value of the z-component of the qubit state vector, $\sum_t \langle\Psi(t)|\hat{\sigma}_z|\Psi(t)\rangle/\Delta T$, during the readout pulse with the width $\Delta T = 500$ ns. The influence of the feedback error is simulated by inverting the outcome k of the readout with the experimentally obtained error probability (Fig. 6).

SUPPLEMENTARY REFERENCES

[1] Funo, K., Murashita, Y. & Ueda, M. Quantum nonequilibrium equalities with absolute irreversibility. *New J. Phys.* **17**, 075005 (2015).

[2] Tasaki, H. Jarzynski relations for quantum systems and some applications. *arXiv: cond-mat/0009244* (2000).

[3] Funo, K., Watanabe, Y. & Ueda, M. Integral quantum fluctuation theorems under measurement and feedback control. *Phys. Rev. E* **88**, 052121 (2013).

[4] Yamamoto, T. *et al.* Flux-driven josephson parametric amplifier. *Appl. Phys. Lett.* **93**, – (2008).

[5] Koch, J. *et al.* Charge-insensitive qubit design derived from the Cooper pair box. *Phys. Rev. A* **76**, 042319 (2007).

[6] Blais, A., Huang, R.-S., Wallraff, A., Girvin, S. M. & Schoelkopf, R. J. Cavity quantum electrodynamics for superconducting electrical circuits: An architecture for quantum computation. *Phys. Rev. A* **69**, 062320 (2004).

[7] Dalibard, J., Castin, Y. & Mølmer, K. Wave-function approach to dissipative processes in quantum optics. *Phys. Rev. Lett.* **68**, 580–583 (1992).

[8] Mølmer, K., Castin, Y. & Dalibard, J. Monte carlo wave-function method in quantum optics. *J. Opt. Soc. Am. B* **10**, 524–538 (1993).

[9] Gardiner, C. & Zoller, P. *Quantum Noise* (Springer, 2004).
